# Bequests to Hospitals

**Published:** 1910-02-26

**Authors:** 


### BEQUESTS TO HOSPITALS.

While hospital authorities are always glad to appear in the *rôle* of legatees, there are some bequests which give rise to considerable trouble, and involve costly litigation. Where a legacy is "open," that is to say, where money is left to a hospital generally, difficulty is not likely to arise; but where the testator desires to ear-mark his bequest by giving money for a specific purpose, he may fall into error if he does not consult his solicitor. Lawyers are said to drink the health of the man who makes his own will! Why they should drink his health is matter for speculation, since his early demise may enhance the prospect of expensive litigation; but the hospital which finds a large part of its legacy swallowed up in costs feels anything but grateful. As an example of the difficulties to which the careless preparation of a will may give rise, mention may be made of a case in which a testator gave his residue as follows: One half to the Foundling Hospital and the other half to the Lying-in Hospital, and if there should be more than one of the latter, then to such of them as the executor should appoint. He died without having appointed an executor. Lord Thurlow upheld the gift of the second half, and directed an inquiry as to which of the Lying-in Hospitals it should be paid. It is an old case; but we wonder what the costs of the litigation amounted to. The evils which may follow upon a bequest for a specific purpose are illustrated in a case which recently came before Mr. Justice Swinfen Eady. A man had left the sum of £1,000 to a hospital to found a bed to be called "the Eleanor bed." The hospital authorities applied the money partly in paying off debt and partly to general purposes. The Attorney-General then brought an action for a declaration as to the true meaning of the bequest, and that the defendants should be ordered to pay £1,000 to the Official Trustee of Charitable Funds for investment. His Lordship held that where a sum is bequeathed to a hospital to enable a bed to bear a specified name to be provided, the particular word used by the testator—whether "found," "establish," "endow," or the like—is immaterial. If the intention to establish the bed in perpetuity can be gathered from the will the amount bequeathed must be invested, and a bed maintained out of the income so far as it will go. It is not, however, necessary that there should be a new bed if the specified name can be given to an existing bed in the hospital.

Another recent case relating to bequests to hospitals came before Mr. Justice Joyce in November. There the question arose as to the meaning of a deed by which the income of certain funds was to be applied "for the benefit of the Buchanan Hospital, Springfield Road, St. Leonard's-on-Sea, or such other medical charity or charities of any kind, school, or teaching whatsoever, and partly or exclusively to one or other of such objects as the trustees might in their uncontrolled discretion from time to time determine." The point was whether under such a deed some part of the funds might be devoted to a medical charity or charities situate or carrying on operations in Scotland, that being the country of origin of the person from whom the money originally came. His Lordship held that where there was a scheme of this kind in general terms without any reference to foreign countries, it must be taken to mean that the charity must be administered and the trusts carried into effect within the jurisdiction of the Court by which the scheme was sanctioned.

Having regard to the difficulties adumbrated by the cases just referred to, would it not be justifiable for those who know that money is about to be left to a hospital to hint a wish as to the form of bequest? A suitable form is often attached to the printed circular which is sent out for subscriptions. It may not be proper to look a gift horse in the mouth; but to show the donor's groom the way to your stable is surely justifiable.

